# Functional characteristics of the *Staphylococcus aureus* δ-toxin allelic variant G10S

**DOI:** 10.1038/srep18023

**Published:** 2015-12-10

**Authors:** Gordon Y. C. Cheung, Anthony J. Yeh, Dorothee Kretschmer, Anthony C. Duong, Kwame Tuffuor, Chih-Lung Fu, Hwang-Soo Joo, Binh A. Diep, Min Li, Yuumi Nakamura, Gabriel Nunez, Andreas Peschel, Michael Otto

**Affiliations:** 1 Pathogen Molecular Genetics Section, Laboratory of Bacteriology, National Institute of Allergy and Infectious Diseases, The National Institutes of Health, Bethesda, MD 20892, United States of America; 2 Cellular and Molecular Microbiology Division, Interfaculty Institute of Microbiology and Infection Medicine, University of Tübingen, Tübingen 72076, Germany; 3 Division of Infectious Diseases, Department of Medicine, University of California, San Francisco, San Francisco, CA 94110, United States of America; 4 Department of Laboratory Medicine, Renji Hospital, School of Medicine, Shanghai Jiaotong University, Shanghai 200127, China; 5 Department of Dermatology, Chiba University Graduate School of Medicine, Chiba 2608670, Japan; 6 Department of Pathology and Comprehensive Cancer Center, University of Michigan Medical School, Ann Arbor, MI 48109, United States of America

## Abstract

*Staphylococcus aureus* δ-toxin is a member of the phenol-soluble modulin (PSM) peptide family. PSMs have multiple functions in staphylococcal pathogenesis; for example, they lyse red and white blood cells and trigger inflammatory responses. Compared to other PSMs, δ-toxin is usually more strongly expressed but has only moderate cytolytic capacities. The amino acid sequences of *S. aureus* PSMs are well conserved with two exceptions, one of which is the δ-toxin allelic variant G10S. This variant is a characteristic of the subspecies *S. argenteus* and *S. aureus* sequence types ST1 and ST59, the latter representing the most frequent cause of community-associated infections in Asia. δ-toxin G10S and strains expressing that variant from plasmids or the genome had significantly reduced cytolytic and pro-inflammatory capacities, including in a strain background with pronounced production of other PSMs. However, in murine infection models, isogenic strains expressing the two δ-toxin variants did not cause measurable differences in disease severity. Our findings indicate that the widespread G10S allelic variation of the δ-toxin locus has a significant impact on key pathogenesis mechanisms, but more potent members of the PSM peptide family may overshadow that impact *in vivo*.

Many members of the genus *Staphylococcus* are important human pathogens. *Staphylococcus aureus* in particular causes a multitude of frequently severe and life-threatening diseases, with acute disease promoted by a series of secreted toxins and other virulence determinants^1^,^2^. In addition, *S. aureus* and coagulase-negative staphylococci, such as *Staphylococcus epidermidis*, are a premier cause of hospital-associated infections on indwelling medical devices^3^.

While virulence of *S. aureus* is clearly multi-factorial, the phenol-soluble modulin (PSM) peptide family has recently been identified as a key contributor to infection with highly virulent *S. aureus* strains, such as community-associated methicillin-resistant *S. aureus* (CA-MRSA)^4^,^5^,^6^. PSMs are short peptides that are under strict regulation by the Agr quorum-sensing system^7^ and secreted by a dedicated transporter without a signal peptide^8^. They can be grouped into the shorter (~25 amino acid) α-type peptides, which in *S. aureus* comprise the PSMα1 through PSMα4 peptides and the δ-toxin, and the longer (~45 amino acid) β-type peptides, of which two (PSMβ1, PSMβ2) are produced by *S. aureus*^4^. An additional PSM peptide, PSM-mec, is encoded on the meticillin resistance cassette SCC*mec* and only produced by a certain group of meticillin-resistant *S. aureus*^7^. The PSMα peptides in particular strongly impact the capacity of CA-MRSA strains to lyse human neutrophils and other cell types, and promote skin infection and bacteraemia^5^. Less virulent hospital-associated strains characteristically produce smaller amounts of those peptide toxins^5^,^9^.

In addition to their cytolytic potential, PSM peptides promote inflammatory responses by activating the formyl peptide receptor 2 (FPR2)^10^. Furthermore, they contribute to biofilm structuring, detachment, and the systemic dissemination of biofilm-associated infection^11^,^12^. Moreover, some PSMs may exhibit antimicrobial functions, for example toward *Streptococcus pyogenes*^13^,^14^.

The δ-toxin is the member of the PSM family that has been known for the longest time. Originally believed to be a large protein, it is in fact a peptide that tends to oligomerize^15^,^16^. It is encoded within RNAIII, the regulatory molecule of the accessory gene regulator (Agr) quorum-sensing system^17^. Notably, despite frequent incorrect annotation in data banks, the δ-toxin does not contain a signal peptide, but is a 26 amino acid long peptide that – as the other members of the PSM family – is secreted as the primary translation product with an N-terminal N-formyl methionine^8^. Variants of the δ-toxin with often highly similar peptide sequences are present in many staphylococcal species^18^. The δ-toxin is usually the most strongly produced PSM peptide and in many strains by far the most abundant secreted protein^5^,^8^,^19^.

While known for many years, the specific functions of the δ-toxin in pathogenesis and physiology have remained largely obscure. Previous correlative analyses between pathogenesis phenotypes and δ-toxin production hardly allow conclusions on the specific function of δ-toxin, because δ-toxin is Agr-regulated in the same way as many other toxins, including, most notably, PSMs^7^,^17^. However, the discovery of PSMs also triggered a more detailed investigation of the contribution of δ-toxin to pathogenesis. To that end, isogenic *hld* mutants were constructed with start codon mutations, abolishing translation but maintaining the function of RNAIII^5^.

Compared to other α-type PSMs, δ-toxin has generally more moderate potency^5^. It is somewhat cytolytic to neutrophils and erythrocytes and has moderate capacity to stimulate FPR2^5^,^10^. Accordingly, an isogenic δ-toxin production mutant (genomic *hld* start codon mutant) in the community-associated meticillin-resistant *S. aureus* (CA-MRSA) strain MW2 only showed a slight impact on disease progression in a murine bacteraemia model, especially when compared to the strong contribution of the *psm*α locus (encoding PSMα1 through PSMα4), and no impact on skin infection in the USA300 (LAC) CA-MRSA background^5^. The first truly δ-toxin-specific function has only recently been discovered. The δ-toxin, but not other PSMs, triggers mast cell degranulation and thus has a significant contribution to the development of the skin disease, atopic dermatitis^20^.

Here we analysed the effect that the only widespread allelic variant of the δ-toxin locus, δ-toxin G10S, has on cytolytic and pro-inflammatory properties, and whether the changed properties that we found translate to a significant impact on disease progression in animal infection models.

## Results

### The G10S allelic variant of δ-toxin is a characteristic of ST1 and ST59 isolates

**Figure 1:**
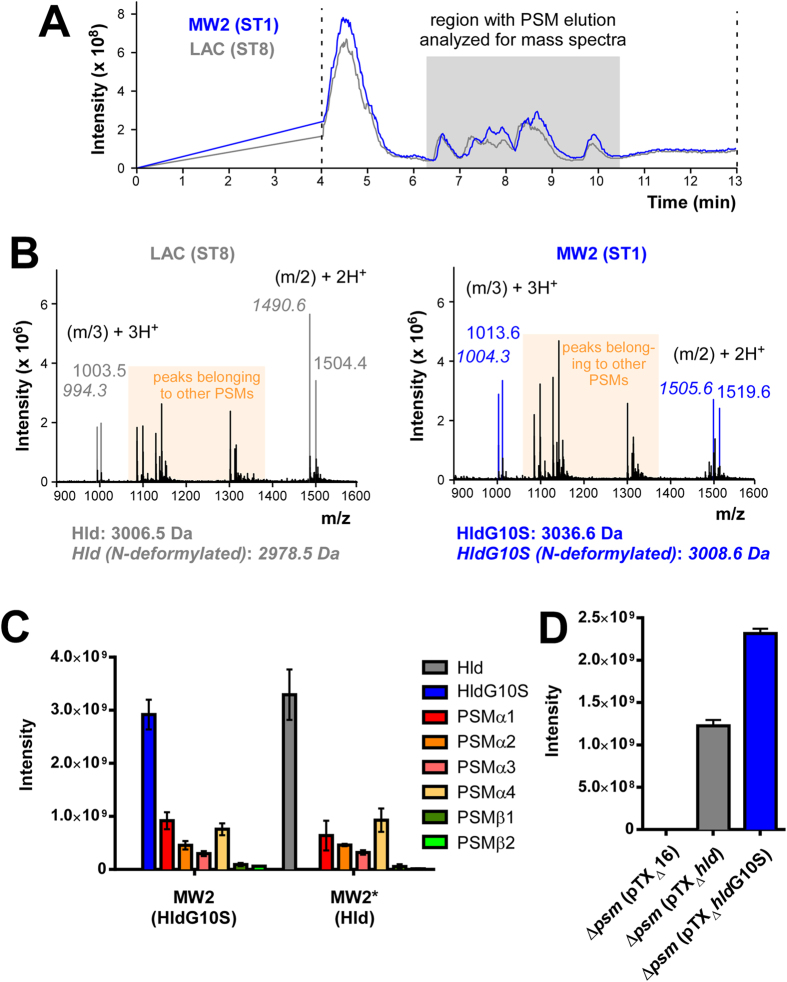
Detection of HldG10S and construction of allelic variant expression strains. (**A**) Total ion chromatograms (TICs) of reversed phase-HPLC/MS of 8-h MW2 (expressing HldG10S) and LAC (expressing Hld) culture filtrates grown in TSB. TICs were recorded between 4 and 13 min of elution. (**B**) Average mass spectra of the PSM elution range (see gray box in panel **A**). Theoretical masses of Hld and HldG10S in their N-formylated and N-deformylated forms are given below the spectra. (**C**) PSM production in the genetically altered strain MW2* (expressing Hld) in comparison to wild-type MW2. (**D**) Comparison of Hld/HldG10S production in LACΔαβ*hld* with expression and control plasmids. (**C**,**D**) Values are from triplicate experiments.

The analysis of available *S. aureus* genomes, as well as high pressure liquid chromatography/mass spectrometry (HPLC/MS) analysis of many *S. aureus* culture filtrates that we performed over the years, revealed the presence of two main allelic variants among *S. aureus* PSM peptides. One is the PSMα3 variant PSMα3N22Y that is characteristically present in clonal complex (CC) 30 and on which we reported earlier^21^. The other is due to a non-synonymous mutation in the *hld* gene (from the glycine codon GGT to the serine codon AGT), leading to a δ-toxin (Hld) peptide with a serine instead of glycine at position 10 (HldG10S) (Fig. 1A,B). This allelic variant is found in 41 published genomes or genome projects, including notable ST1 and ST59 strains, such as, ST59 strains M013^22^, SA268^23^, SA40^24^, and SA957^24^, and ST1 strains MW2^25^ and MSSA476^26^, in addition to the new sub-species “*Staphylococcus argenteus*” [*S. aureus* clonal complex (CC) 75]^27^. ST59 is the most frequent lineage causing methicillin-susceptible and methicillin-resistant community-associated (CA) infections in China and adjacent Asian countries^28^. We analysed a series of ST59 isolates (8 from China and 13 from San Francisco), all of which showed the characteristic mass of the G10S variant of δ-toxin upon HPLC/MS analysis, indicating that this variant is a characteristic of that successful lineage. Of note, we never found HldG10S in many *S. aureus* isolates that we analysed by HPLC/MS over recent years other than those of the ST1 or ST59 lineages. (We did not analyse *S. argenteus* or other CC75 isolates by HPLC/MS.) Together, these findings indicate that HldG10S is characteristic of a genetic subset of *S. aureus* (including ST1 and ST59) and the subspecies *S. argenteus*.

### HldG10S has decreased cytolytic capacity

At micromolar concentrations, many α-type PSMs are strongly cytolytic towards many types of eukaryotic cells, which include human erythrocytes and neutrophils^5^,^19^. This mechanism of action is believed to play an important role in the progression of staphylococcal disease^6^. To determine if there are differences in the lytic activities between Hld and HldG10S, we compared (i) synthetic peptides, (ii) culture supernatants of *S. aureus* strains expressing those peptides from plasmids in a PSM-negative background (strain USA300 LAC with all *psm* genes deleted and translation of *hld* abolished, LAC Δ*αβhld* or Δ*psm*), or (iii) culture supernatant of the ST1 strain MW2 (HldG10S) in comparison to that from an isogenic mutant that was engineered to express “normal” Hld from the genome. The genetically altered MW2 strain, MW2*, produced amounts of Hld that were equal to those of HldG10S produced by the isogenic strain MW2 (Fig. 1C). Furthermore, production of other PSMs was unaltered. However, when the δ-toxin variants were expressed from plasmids, we observed a somewhat stronger production of HldG10S than Hld from the same strain and plasmid background (Fig. 1D).

**Figure 2:**
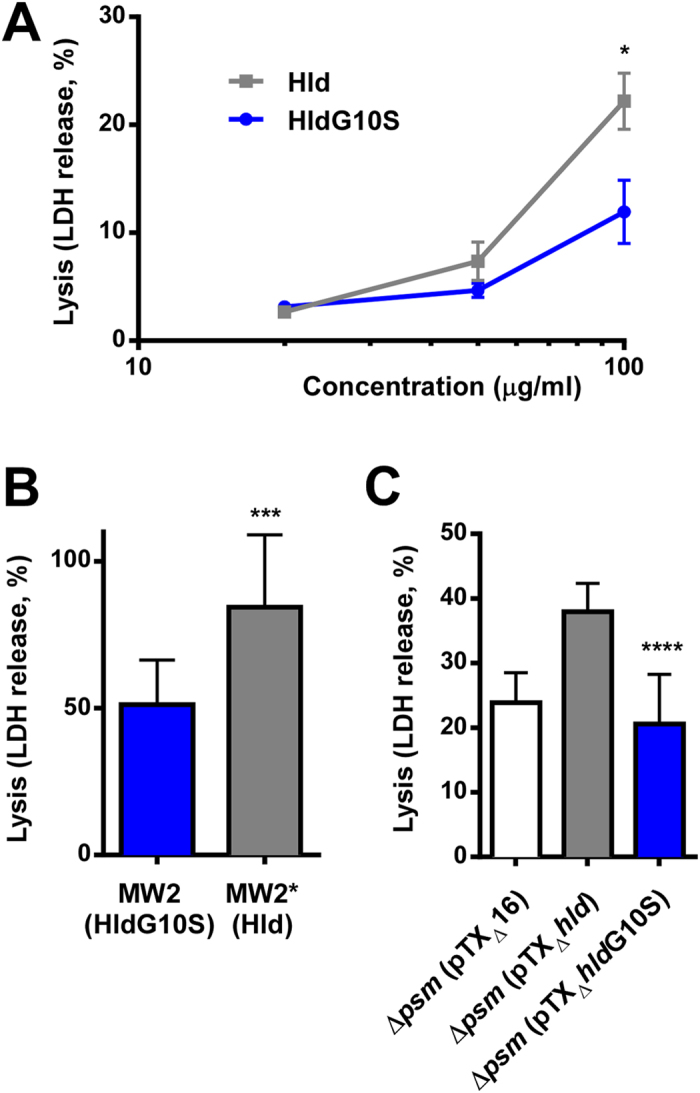
Impact of the Hld allelic variation on neutrophil lysis. Lysis of human neutrophils was measured by release of LDH with synthetic peptides (**A**), culture filtrates of isogenic strains expressing Hld or HldG10S from the genome (**B**), and culture filtrates from the PSM-negative strain LACΔαβ*hld* expressing Hld or HldG10S from plasmids (**C**). All values are from triplicate experiments. *p < 0.05; ***p < 0.001; ****p < 0.0001.

**Figure 3:**
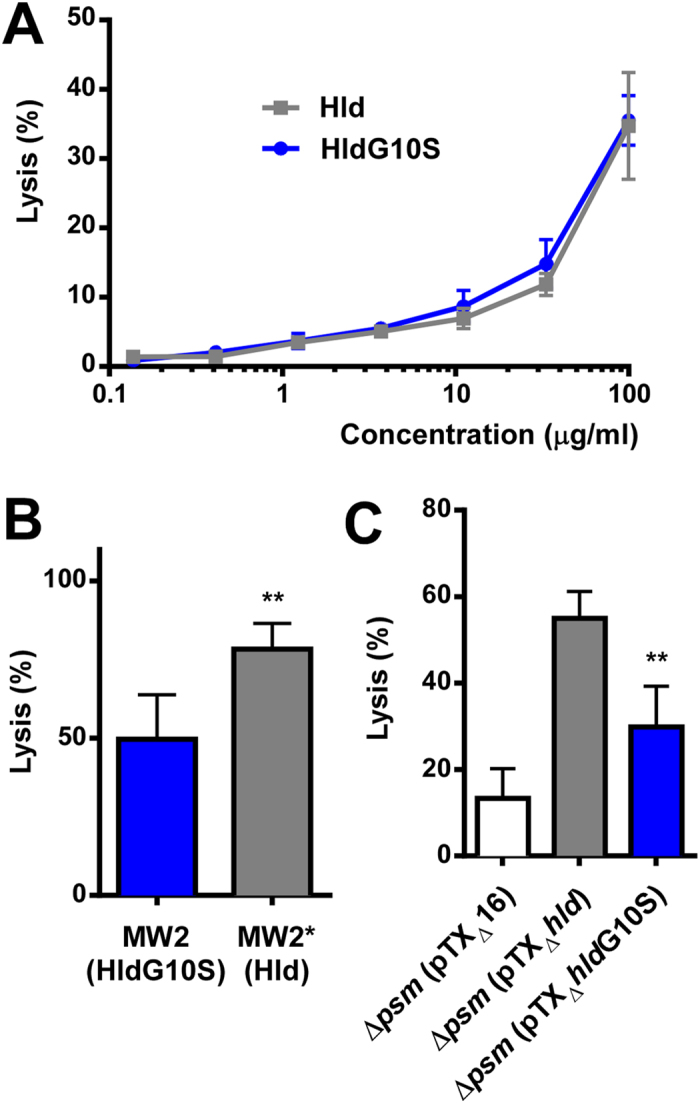
Impact of the Hld allelic variation on erythrocyte lysis. Lysis of human erythrocytes was measured by haemolysis assays with synthetic peptides (**A**), culture filtrates of isogenic strains expressing Hld or HldG10S from the genome (**B**), and culture filtrates from the PSM-negative strain LACΔαβ*hld* expressing Hld or HldG10S from plasmids (**C**). All values are from triplicate experiments. **p < 0.01.

Lysis of human neutrophils was significantly decreased with synthetic HldG10S (Fig. 2A) and with culture filtrates of strains expressing that peptide from a plasmid (Fig. 2B) or the genome (Fig. 2C) as compared to Hld and isogenic strains expressing Hld. Similarly, lysis of human erythrocytes was significantly decreased with culture filtrates containing HldG10S (Fig. 3B,C). However, there was no significant difference using synthetic HldG10S peptide (Fig. 3A), possibly indicating that the difference in erythrocyte lysis observed with culture filtrates is due to synergistic haemolytic activity of δ-toxin with other toxins. Together, these findings show that the cytolytic properties of strains expressing HldG10S are significantly lower than of those expressing Hld, which at least in the case of lytic activity toward neutrophils is due to differential cytolytic properties of the Hld and HldG10S peptides.

### HldG10S has decreased chemotactic activity

**Figure 4:**
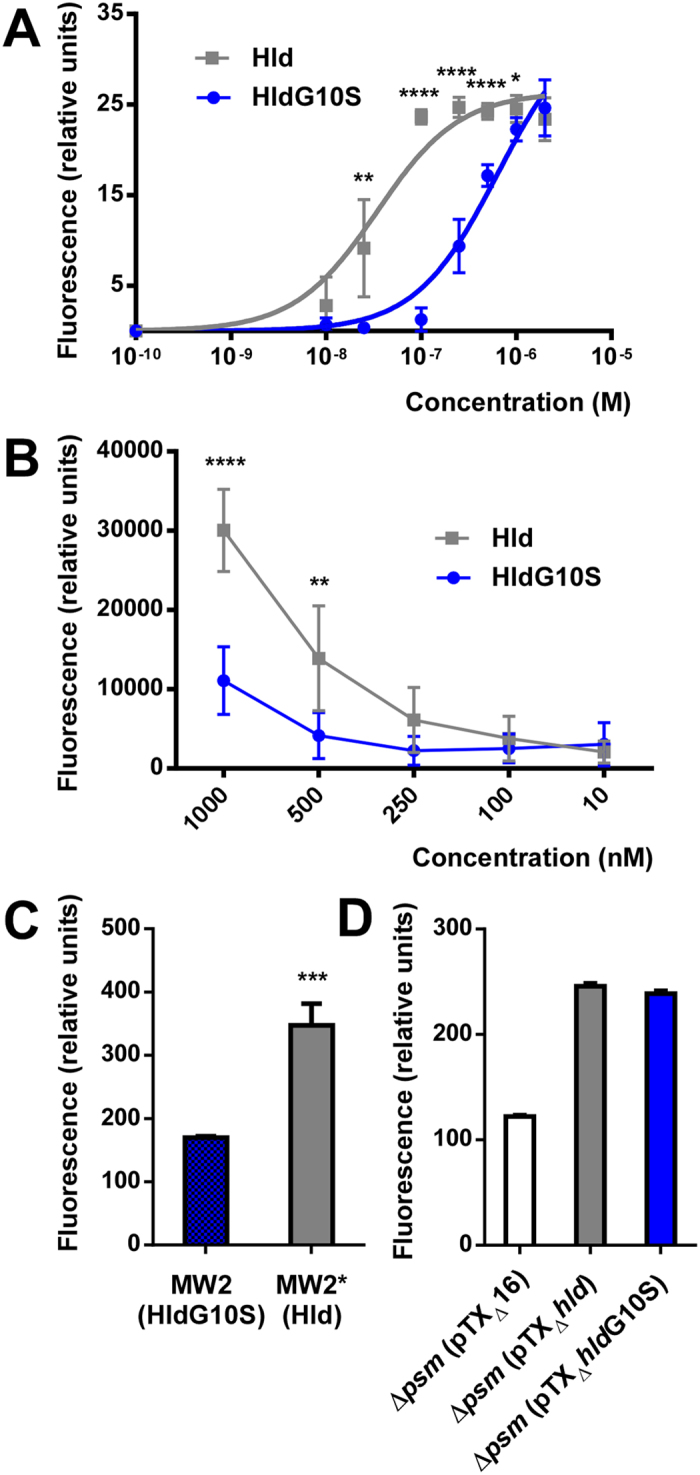
Impact of the Hld allelic variation on calcium flux and chemotaxis of human neutrophils. (**A**) Calcium flux with synthetic peptides. (**B**) Chemotaxis with synthetic peptides. (**C**,**D**) Chemotaxis with culture filtrates of isogenic strains expressing Hld or HldG10S from the genome, and culture filtrates from the PSM-negative strain LACΔαβ*hld* expressing Hld or HldG10S from plasmids, respectively). All values are from triplicate experiments. *p < 0.05; **p < 0.01; ***p < 0.001; ****p < 0.0001.

Calcium ion influx has been used as readout for the pro-inflammatory capacity PSMs exert on human neutrophils^10^. Ca^2+^ influx was significantly decreased with synthetic HldG10S compared to Hld, indicating significantly different capacities of Hld and HldG10S to stimulate FPR2 (Fig. 4A).

Chemotactic activity toward neutrophils is one of the most prominent pro-inflammatory functions that PSMs trigger via interaction with FPR2^10^. Therefore, we measured chemotaxis of human neutrophils to investigate whether the HldG10S allelic variant has different pro-inflammatory capacity than Hld. Synthetic HldG10S and culture filtrates of MW2 (expressing HldG10S) had significantly decreased chemotactic activity compared to Hld and culture filtrates of MW2* (expressing Hld), respectively (Fig. 4B–D). Activity was not changed comparing culture filtrates of the plasmid-harbouring strains, which, however, may be due to the somewhat higher production of HldG10S as compared to Hld that we observed with those constructs (Fig. 1C). These findings show that the FPR2-mediated pro-inflammatory capacity of HldG10S is lower than that of Hld.

### HldG10S does not cause a change in mast cell degranulation, biofilm formation, cell spreading or antimicrobial activity

**Figure 5:**
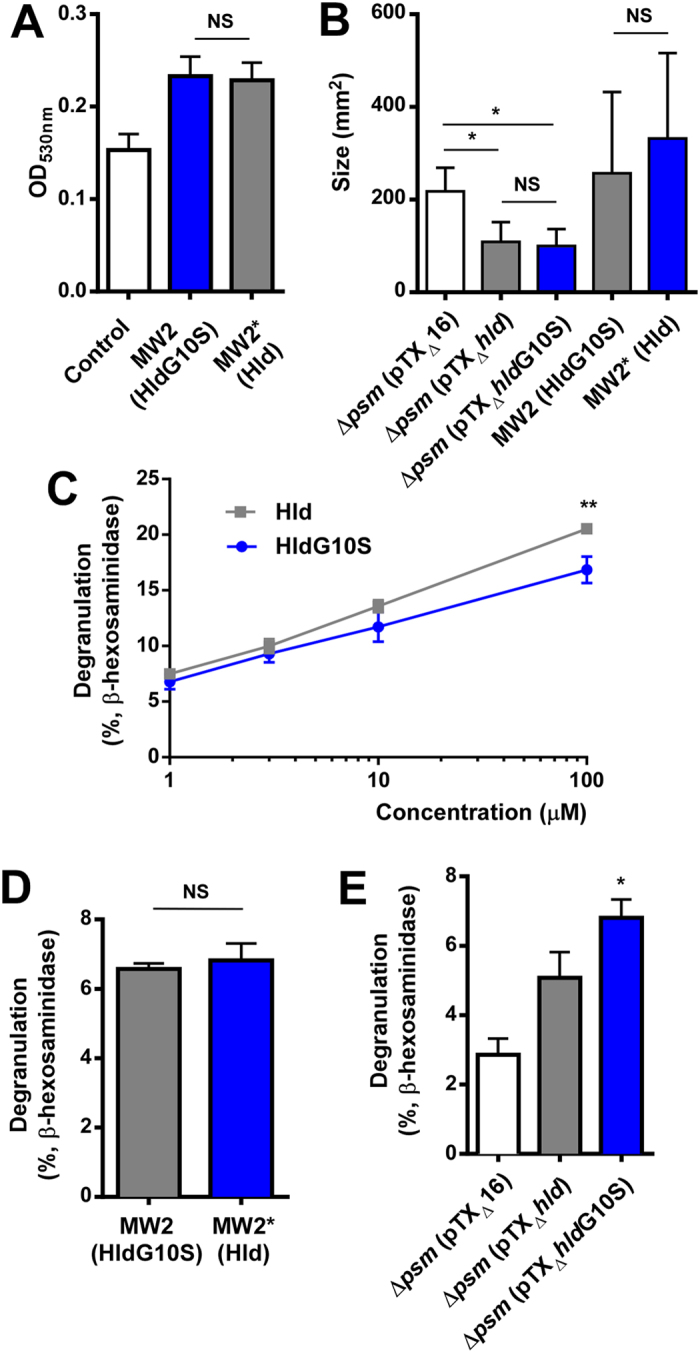
Impact of the Hld allelic variation on biofilm formation and mast cell degranulation. (**A**) Biofilm formation in microtiter plates and (**B**) cell spreading on agar surface by isogenic strains expressing Hld or HldG10S from the genome. (**C**) Mast cell degranulation by synthetic peptides. (**D**,**E**) Mast cell degranulation with culture filtrates of isogenic strains expressing Hld or HldG10S from the genome, and culture filtrates from the PSM-negative strain LACΔαβ*hld* expressing Hld or HldG10S from plasmids, respectively. All values are from triplicate experiments. *p < 0.05; NS, not significant.

All PSMs including δ-toxin impact biofilm structuring and detachment in a way that leads to extended biofilm formation in their absence^11^,^29^. Therefore, we tested whether there is a difference in biofilm formation between the isogenic MW2 strain pair expressing Hld versus HldG10S. However, semi-quantitative biofilm assays did not reveal differences in biofilm formation due to the δ-toxin allelic variation (Fig. 5A).

Some PSMs, including δ-toxin, have antimicrobial activities, in particular against *Steptococcus pyogenes*^13^,^14^. However, there no antimicrobial activity was detected with considerable amounts of either Hld or HldG10S (data not shown).

PSMs are believed to contribute to the commensal lifestyle of staphylococci by promoting emulsification of nutrients and cell spreading on surfaces^30^,^31^. In the case of δ-toxin, contradictory results have been obtained depending on whether synthetic peptide was used or δ-toxin-expressing strains^30^,^32^. Our results using isogenic δ-toxin-expressing and control strains reflected those by Omae *et al.*^32^, inasmuch as the δ-toxin expressing strains spread less than the control strain. However, no differences in cell spreading were observed comparing MW2 (HldG10S) and MW2* (Hld) or isogenic PSM-free strains complemented with plasmids expressing Hld or HldG10S (Fig. 5B).

Recently, δ-toxin was found to be a key factor promoting pathogenesis of atopic dermatitis by causing degranulation of mast cells^20^. Differences in mast cell degranulation comparing HldG10S and Hld peptides were very small, and absent comparing MW2 (HldG10S) and MW2* (Hld) culture filtrates (Fig. 5C,D). The somewhat higher degranulation values by culture filtrates of the strain expressing HldG10S from a plasmid, as compared to the strain expressing Hld (Fig. 5E), were in the range to be explained by the abovementioned differences in expression levels (Fig. 1C). These findings indicate that mast cell degranulation, biofilm formation, cell spreading, and antimicrobial activity are not impacted significantly by the HldG10S allelic variation.

### The HldG10S variation does not significantly impact *S. aureus* experimental infection

**Figure 6:**
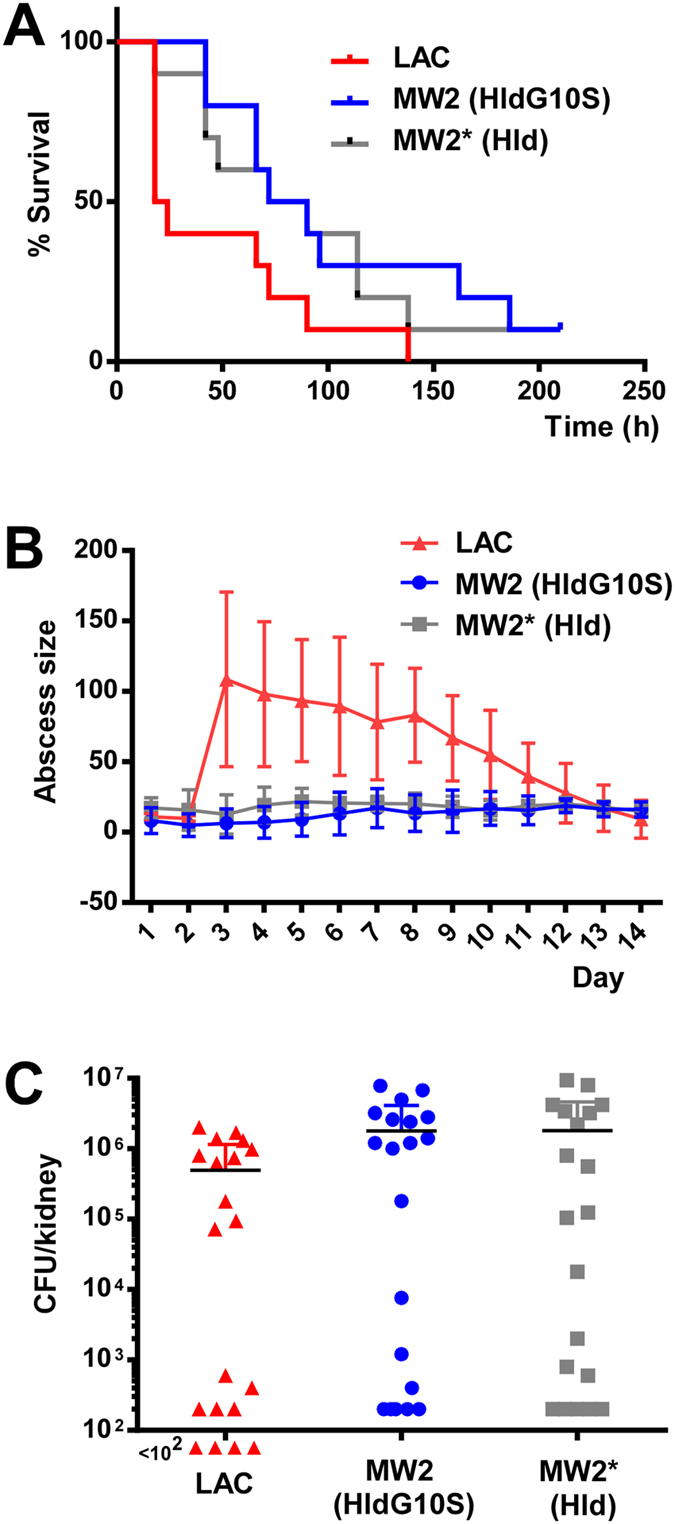
Impact of the Hld allelic variation on virulence in murine infection models. (**A**) Bacteraemia model, survival rates. (**B**) Abscess model, abscess sizes. Note strain LAC produced open lesions, while MW2 and MW2* produced closed abscesses. (**C**) Renal abscess model, CFUs in one kidney. With strain LAC, 4 values were 0; these are depicted below the x axis.

Among widespread *S. aureus* toxins, those with the most prominent impact on key *S. aureus* infection types like skin and blood infection, are PSMα peptides and α-toxin^5^,^33^,^34^,^35^. Nevertheless, we previously showed that abolishing translation of δ-toxin in strain MW2 also has a significant, yet less pronounced, impact on mortality in a murine bacteraemia model^5^. Therefore, we analysed whether the HldG10S variation present in strain MW2 has an impact on disease in murine blood (Fig. 6A) and skin (Fig. 6B) infection models as compared to the isogenic MW2* strain altered to express Hld. Furthermore, we included the sensitive renal abscess model to best detect possibly only subtle differences (Fig. 6C). We did not detect significant differences in disease severity between mice infected with the MW2 or MW2* strains in any of the three infection models. Interestingly, strain MW2 was about as virulent in the renal abscess and blood infection models as strain LAC (USA300), but much less virulent in the skin infection model.

## Discussion

Over the last years, PSMs have been increasingly recognized as important factors affecting both the commensal and infectious lifestyles of staphylococci^4^,^6^. Acute virulence of *S. aureus* in particular is to a large extent defined by production of the cytolytic PSMα peptides^5^,^36^,^37^. Cytolytic and pro-inflammatory capacities of the δ-toxin are in the range of other α-type PSMs, but fall short of reaching the strong potencies of, for example, PSMα3 of *S. aureus* or PSMδ of *S. epidermidis*^5^,^19^. Nevertheless, δ-toxin has been shown to impact *S. aureus* blood infection even in the background of a strain producing considerable levels of more potent PSMs^5^. Furthermore, it has recently been demonstrated to be critical for the pathogenesis of atopic dermatitis by degranulation of mast cells^20^, a feature not observed with other *S. aureus* PSMs.

Here we analysed available genome sequences and performed HPLC/MS, showing that *S. aureus* ST1 and ST59 strains, and *S. argenteus*, produce an allelic variant of δ-toxin, HldG10S. According to our analyses, HldG10S represents the only widespread allelic variant of a PSM peptide in *S. aureus* other than the PSMα3N22Y variant present in CC30 strains^21^.

HldG10S generally had decreased aggressive potencies, such as lower cytolytic activity and reduced chemotactic activity toward human neutrophils. Notably, these differences were also observed in an isogenic strain pair producing either of the two variants in a background with otherwise strong PSM production^5^, indicating that the allelic variation may influence pathogenesis phenotypes *in vivo*. However, despite the key role of neutrophils in the defence against *S. aureus* infection^38^, we could not detect differences between disease phenotypes elicited by this isogenic strain pair in several murine infection models. We believe that this is due to the generally limited impact of δ-toxin on *S. aureus* infection as compared to other, more potent PSMs^5^ and other toxins such as α-toxin^35^,^39^. Furthermore, the δ-toxin allelic variants did not differentially impact mast cell degranulation as the only known pathogenesis mechanism that is specifically influenced by δ-toxin among PSMs^20^. This indicates that differences in the capacities of *S. aureus* strains to stimulate inflammation during atopic dermatitis are not linked to allelic variation of δ-toxin.

Interestingly, our haemolysis data suggest that the differential impact on erythrocyte lysis between the two δ-toxin variants may be due to synergistic activities rather than differences in the lytic activities of δ-toxin itself, a phenomenon that remains to be explored further. Possible interaction partners of δ-toxin in the used MW2 strain background are α-toxin and leukotoxins, such as the Panton-Valentine leukocidin (PVL), for which synergistic activity with other PSMs has been described^40^. Notably, strain MW2 does not produce β-toxin, a protein known to show synergistic hemolysis with δ-toxin^41^,^42^.

In conclusion, our study shows that the allelic variant of the *S. aureus* δ-toxin HldG10S is a characteristic of specific *S. aureus* lineages, among which ST59 is the most epidemiologically important. It significantly impacts cytolytic and pro-inflammatory properties, also when tested in a natural strain background with pronounced general production of PSMs. While these differences did not translate into measurable differences in the tested animal infection models, the differential activities of HldG10S may well influence disease progression under different conditions or in strains with lower production of other PSMs.

## Materials and Methods

### Ethics Statement

The animal protocol (LB1E) was reviewed and approved by the Animal Care and Use Committee at the NIAID, NIH, according to the animal welfare act of the United States (7 U.S.C. 2131 et. seq.). All mouse experiments were performed at the animal care facility of the NIAID, Building 33, in accordance with approved guidelines. All animals were euthanized by CO_2_ at the end of the studies. Human neutrophils were isolated from blood obtained under approved protocols at the NIH Blood Bank or with a protocol (633/2012BO2) approved by the Institutional Review Board for Human Subjects, NIAID, NIH and the University of Tübingen, Germany. Informed written consent was obtained from all volunteers.

### Bacterial strains, plasmids and culture conditions

The bacterial strains used in this study are listed in Table 1. *S. aureus* strains MW2 and LAC are CA-MRSA strains of pulsed-field types USA400 and USA300 isolates, respectively.

*S. aureus* was grown in tryptic soy broth (TSB) at 37 °C with shaking at 180 rpm with the addition of 10 μg/ml chloramphenicol, 100 μg/ml ampicillin or 12.5 μg/ml tetracycline when required. Culture supernatants were collected after 18 h post inoculation from 50-ml *S. aureus* cultures grown in 125 ml baffled flasks with shaking at 180 rpm at 37 °C. The culture supernatants were filtered through PES filters (0.2 μm pore size, Millipore) and used fresh or stored at −20 °C until needed. For all animal experiments, bacteria were inoculated from a pre-culture and grown to mid-exponential growth phase (~2 h), harvested, washed, and diluted with sterile PBS.

### Peptides

N-terminal N-formyl methionine synthetic Hld (fMAQDIISTIGDLVKWIIDTVNKFTKK) and HldG10S (fMAQDIISTISDLVKWIIDTVNKFTKK) peptides were obtained from commercial vendors at a purity of >95%. Dimethyl sulfoxide was used to prepare stock solutions of peptides at 10 mg/ml. Peptides were subsequently diluted in RPMI 1640 (Gibco) for neutrophil or Dulbecco’s phosphate-buffered saline (DPBS) (Sigma) for haemolysis assays.

### Plasmid construction and allelic replacement

A mutation was introduced into strain MW2 to change the serine residue to a glycine residue at position 10 (MW2*). This was accomplished using the allelic replacement method with plasmid pKOR1^43^. Template DNA from strain LAC was used to create DNA fragments for the allelic replacement.

For constitutive *hld* expression, *hld* genes amplified from genomic DNA of strains MW2 or LAC were cloned in plasmid pTX_∆_^5^, which lacks the xylose repressor of pTX15^44^, and transformed in a strain derived from strain LAC that has all *psm* genes deleted and in which translation of the endogenous *hld* gene is abolished by a mutation of the start codon^14^. DNA sequencing was used to confirm the presence and fidelity of cloned inserts in pKOR1 as well as the *hld* S10G mutation in the genome of MW2*.

### Haemolysis

Haemolysis assays were performed as previously described^5^. Briefly, whole blood, which was collected into heparinized tubes from healthy human subjects, were washed twice with ice-cold DPBS and resuspended to a final concentration of 2% (v/v). Equal volumes of PES-filtered supernatants or synthetic peptides were added to the 2% erythrocyte suspension to a total volume of 200 μl in 96 round-bottom plates and incubated for 1 h at 37 °C. The plates were then centrifuged at 233 × g at 4 °C for 5 min, supernatants were collected, and the release of haemoglobin was measured at an optical density of 540 nm.

### Measurement of PSM production by high-pressure liquid chromatography/mass spectrometry (HPLC/MS)

PSM concentrations in culture supernatants were measured using HPLC/MS^45^ with the gradient and flow rate conditions as described in Wang *et al.*^12^.

### Isolation of neutrophils from human venous blood

Human neutrophils were isolated from venous blood of healthy donors as previously described^46^. Briefly, the heparinized blood was incubated with an equal volume of 3% (v/v) dextran (Sigma) for 20 minutes at RT to separate the erythrocytes. The clear supernatant above the erythrocytes was aspirated and leukocytes collected by centrifugation. The cell pellet was resuspended in 0.9% NaCl (Baxter) and layered with Ficoll Hypaque Plus (GE Healthcare). After centrifugation, the supernatant was discarded and the remaining cell pellet was subjected to a brief hypotonic shock with pyrogen-free water. The cells were washed and resuspended in RPMI without phenol red supplemented with 10 mM (4-(2-hydroxyethyl)-1-piperazineethanesulfonic acid, HEPES) to the desired concentration.

### Measurement of neutrophil lysis

The lactate dehydrogenase (LDH) release assay was performed as previously described^19^. Briefly, equal volumes of PES-filtered supernatants or synthetic peptides were added to 1 × 10^6^ neutrophils/ml to a total volume of 200 μl in 96 flat-bottom plates and incubated for 1 h at 37 °C. At the desired times, the plates were then centrifuged at 233 × g at 4 °C for 5 min, supernatants collected, and the release of LDH was measured using the Cytotoxicity Detection Kit (Roche Applied Sciences).

### Measurement of neutrophil chemotaxis and Ca^2+^ flux

Neutrophil chemotaxis was determined using a transwell system (Costar) with analysis of neutrophil migration using fluorescence labelling as previously described^5^. For measurement of Ca^2+^ fluxes, 5 × 10^6^ neutrophils/ml were labelled with a fluorescent dye and analysed with a FACScalibur (Becton Dickinson) as previously described^10^. Measurements of 2,000 events were performed and Ca^2+^ flux was expressed as relative fluorescence corrected for buffer controls.

### Isolation and culturing of mast cells and degranulation assay

Preparation and the purity of mouse bone marrow-derived cultured MCs (BMCMCs) were performed as previously described^20^,^47^. The MC degranulation assay was assessed by β-hexosaminidase assay as previously described^47^. Briefly, 2 × 10^6^ MCs/ml were incubated with or without IgEs (anti-DNP IgE; clone, SPE7; 0.3 μg/ml, anti-TNP IgE; clone, IgE3 and C48-2; 0.5 μg/ml) in RPMI with IL-3 for 15 h.

The cells were resuspended in Tyrode’s buffer (Sigma) at 1 × 10^5^ cells per 100 μl, aliquoted in triplicate into a 96 well U-bottom plate and incubated with EGTA (1 mM, Sigma), LY294002 (100 μM, Sigma), WRW4 (10 μM) and cyclosporine H (10 μM, Alexis Biochemicals) for 30 min, and then stimulated with synthetic Hld peptides or filtered bacterial supernatants for 15 min. Results of various stimuli are given as a relative percentage, where freeze and thaw of total cell culture represents 100%^20^.

### Biofilm formation

Biofilm formation was assayed by semi-quantitative microtiter plate assays as described^48^, with 48-h static incubation of cultures and subsequent safranin staining of biofilms.

### Colony spreading assay

The colony-spreading assay was performed as described by Tsompanidou *et al.*^30^, using isogenic, δ-toxin-expressing or non-expressing strains. The area of cell spreading was measured using the following formula: area = π × radius^2^. The spreading assays were repeated 3 times in triplicate.

### Antimicrobial activity

Antimicrobial activity was analysed by a filter disk diffusion assay on *Micrococcus luteus* and *Streptococcus pyogenes* test plates as described^14^. 200 μg of δ-toxin was spotted on the disks.

### Mouse skin infection model

The mouse skin infection model was performed as previously described^5^. Briefly, ~1 × 10^7^ CFUs of bacteria in 50 μl of PBS were injected subcutaneously into the left flank of 6–8 week-old female Crl:SKH1-hrBR hairless mice (Charles River Laboratories). An electronic caliper was used to measure the length (L) and width (W) of the abscess or lesion caused by the bacterial infection daily for 14 days post-infection. The total size of the abscess was calculated using the formula L × W.

### Sepsis and renal abscess infection models

Six to eight week-old female CD-1 mice (Charles River Laboratories) were infected with ~1 × 10^8^ CFUs or ~1 × 10^7^ CFUs in 100 μl of PBS via the tail vein for the sepsis and renal abscess infection models, respectively. For the sepsis infection model, mouse survival was monitored over the course of 9 days. For the renal abscess infection model, all mice were euthanized by CO_2_ inhalation 3 days post-infection and kidneys were collected and homogenized as described previously^21^. The homogenates were diluted in PBS, plated onto TSB plates, and incubated overnight at 37 °C for CFU counting.

### Statistics

Statistical analysis was performed using Graph Pad Prism version 6.02. For the comparison of two groups, unpaired t-tests were used, for three or more, 1-way or 2-way ANOVA, as appropriate. All error bars depict the standard deviation.

## Additional Information

**How to cite this article**: Cheung, G. Y. C. *et al.* Functional characteristics of the *Staphylococcus aureus* d-toxin allelic variant G10S. *Sci. Rep.* **5**, 18023; doi: 10.1038/srep18023 (2015).

## Figures and Tables

**Table 1:** Strains, plasmids, and oligonucleotides used in this study.

	Remarks/sequence	Source
*Strains - E. coli*		
DH5α	Cloning host	49
*Strains - S. aureus*		
RN4220	Cloning host; derived from NCTC8325-4; r-m+	50
MW2	CA-MRSA strain, USA400, ST1	25
MW2*	MW2 with *hld* gene in the genome altered to express HldG10S	This study
LAC	CA-MRSA strain, USA300, ST8	
LAC Δαβ*hld* (Δ*psm*)	LAC with *psm*α and *psm*β loci deleted and *hld* start codon changed to abolish translation	14
*Plasmids*		
pKOR1-*hld*G10S	Plasmid for exchange of *hld* for *hld*G10S by allelic replacement	This study
pTX_∆_16	Control vector for pTX_Δ_ plasmid series (lipase gene deleted)	5
pTX_∆_*hld*	pTX_Δ_ constitutively expressing Hld	20
pTX_∆_*hld*G10S	pTX_Δ_ constitutively expressing HldG10S	This study
*Oligonucleotides*		
deltavFw	GGGGACAAGTTTGTACAAAAAAGCAGGCTCCCGAATTATTAAGATATCCTGCTC	
deltavRv	GGGGACCACTTTGTACAAGAAAGCTGGGTGAAACTTGAACAATTACAAATAAAACG	
deltavmutFw	GATAATCCATTTTACTAAGTCACCGATTGTTGAAATGA	
deltavmutRv	ACAAGATATCATTTCAACAATCGGTGACTTAGTAAAAT	
